# A case of complete remission by cabozantinib as an end-line treatment for advanced hepatocellular carcinoma

**DOI:** 10.1007/s12328-024-02062-2

**Published:** 2024-12-01

**Authors:** Shuhei Nagashima, Satoshi Kobayashi, Shotaro Tsunoda, Yui Yamachika, Yuichiro Tozuka, Taito Fukushima, Manabu Morimoto, Makoto Ueno, Junji Furuse, Shin Maeda

**Affiliations:** 1 Department of Gastroenterology, Kanagawa Cancer Center, 2-3-2 Nakao Asahi-ku, Yokohama, 241-8515 Japan; 2 Department of Gastroenterology, Yokohama City University Graduate School of Medicine, 3-9, Fukuura, Kanazawa-ku, Yokohama, 236-0004 Japan

**Keywords:** Cabozantinib, Hepatocellular carcinoma, Immune checkpoint inhibitor, Complete remission

## Abstract

Cabozantinib is a multi-kinase inhibitor targeting multiple tyrosine kinases. It improves overall survival and progression-free survival in patients previously treated with sorafenib for advanced hepatocellular carcinoma (HCC) compared to the placebo in the phase 3 CELESTIAL trial. A 71-year-old man presented to our hospital for treatment of HCC with chronic hepatitis C. He was refractory to sorafenib, lenvatinib, regorafenib, and ramucirumab and started atezolizumab and bevacizumab therapy in November 2020. After administering the second cycle on December 10, 2020, the patient was diagnosed with progressive disease in January 2021. Therefore, cabozantinib (60 mg/day) was initiated on January 14, 2021. As the grade 3 aspartate aminotransferase and alanine aminotransferase levels increased, grade 3 anorexia and a decline in performance status were observed in the first week, and cabozantinib was terminated. His performance status and anorexia gradually improved, and contrast-enhanced computed tomography (CT) in June 2021 showed complete remission (CR) according to the modified Response Evaluation Criteria in Solid Tumors. The patient did not show disease progression for 11 months without receiving any treatment for HCC. To the best of our knowledge, this is the first report of CR with cabozantinib in advanced HCC.

## Introduction

Hepatocellular carcinoma (HCC) is the fourth leading cause of cancer-related deaths [1]. For patients with early-stage HCC, treatment options include surgical resection, liver transplantation, radiation therapy, and ablation [2]. However, HCC is often inoperable owing to a late diagnosis, and systemic chemotherapy may be the only treatment option for such patients. Immune checkpoint inhibitors have significantly changed the treatment of HCC in recent years; atezolizumab plus bevacizumab and durvalumab plus tremelimumab improve overall survival compared with sorafenib [3, 4]. Other treatment options include sorafenib, lenvatinib, regorafenib, ramucirumab, and cabozantinib.

Cabozantinib is a multi-kinase inhibitor that targets multiple tyrosine kinases. The phase 3 CELESTIAL trial showed improved efficacy outcomes compared to the placebo in previously sorafenib-treated patients with advanced HCC [5]. However, there are no reports of complete remission (CR) following cabozantinib monotherapy. Here, we report a case of CR according to the modified Response Evaluation Criteria in Solid Tumors (mRECIST) using cabozantinib as an end-line therapy for advanced HCC.

## Case report

A 71-year-old man received interferon therapy for hepatitis C in 2009. Despite the sustained virological response to hepatitis C, HCC developed in S2 and S3 in December 2016, and lateral segmentectomy was performed. In October 2017, lymph node metastases were identified near the hepatic hilum, hepatoduodenal ligament, and peritoneal dissemination node and recurrence were diagnosed. In November 2017, disseminated lesion resection and cholecystectomy were performed, and the patient was referred to another hospital for subsequent treatment. In December 2017, contrast-enhanced computed tomography (CT) revealed that there were still many peritoneal nodules. Sorafenib was started in January 2018, followed by lenvatinib, regorafenib, and ramucirumab. However, the patient did not respond to the treatment, and liver metastasis and peritoneal dissemination were aggravated in August 2020. The patient visited our hospital for further treatment of HCC in September 2020.

**Fig. 1:**
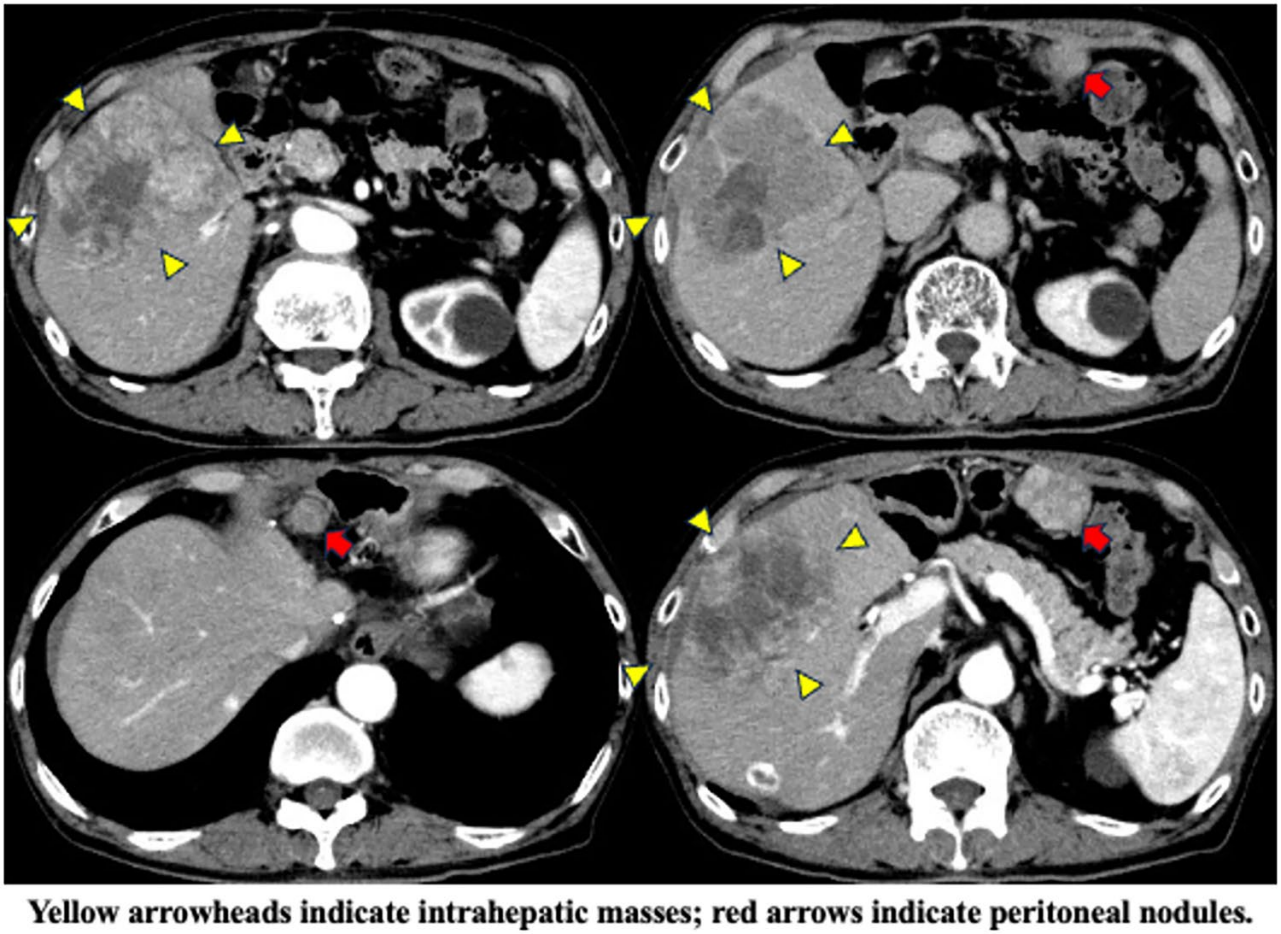
Contrast-enhanced computed tomography upon admission to our hospital

**Fig. 2:**
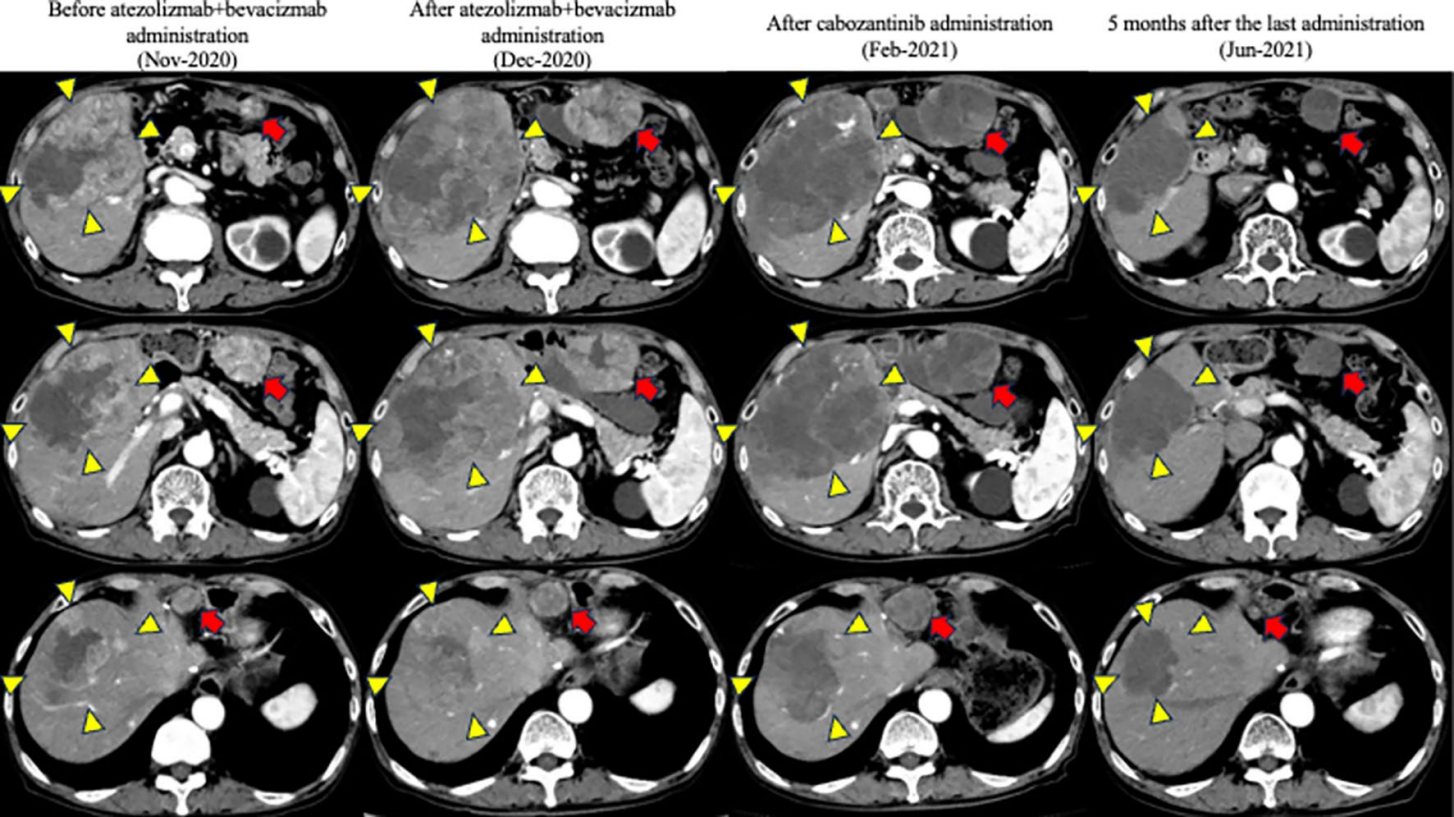
Changes in contrast-enhanced computed tomography image after treatment in our hospital

**Fig. 3:**
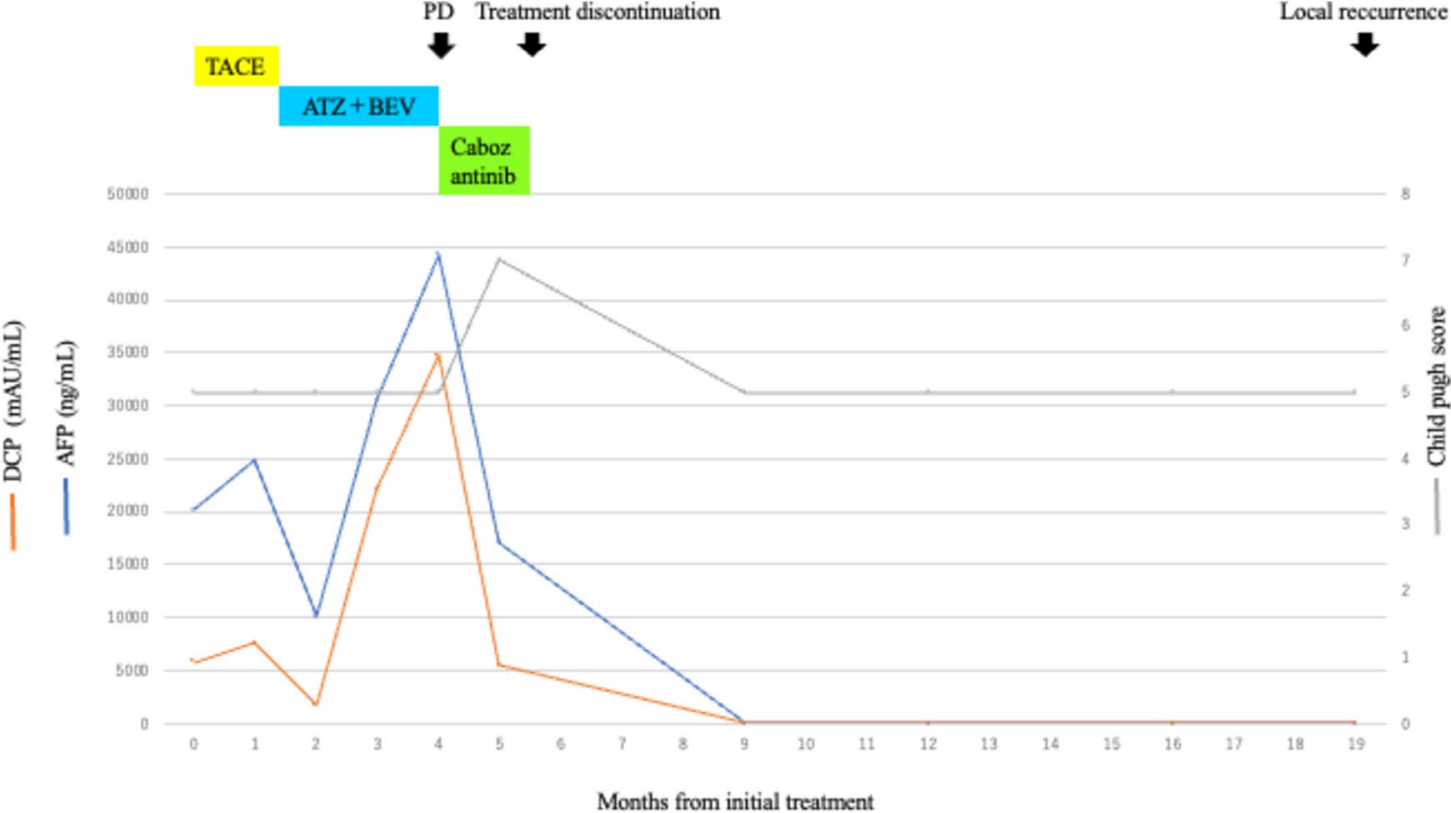
Tumor markers upon admission to our hospital

Contrast-enhanced CT revealed an HCC with a maximum diameter of 10 cm centered on hepatic segments 5 and 8, with a peritoneal dissemination nodule measuring 22 mm in maximum diameter near the hepatoduodenal ligament and a maximum diameter of 35 mm near the omentum (Fig. 1). Minimal ascites was observed on the liver surface, raising suspicion of peritoneal dissemination. On admission, the patient was Child–Pugh class A, with a score of 5. First, we performed transcatheter arterial chemoembolization (TACE) on the intrahepatic lesions to control the tumor and prevent bleeding owing to tumor rupture. Post-treatment CT showed attenuation of enhancement in the intrahepatic lesions; however, intrahepatic lesions and peritoneal dissemination progressed (Fig. 2a). Therefore, we initiated atezolizumab and bevacizumab therapy on November, 2020. There were no obvious treatment-related adverse events; however, progression of all lesions was observed on contrast-enhanced CT after the administration of the second course of atezolizumab and bevacizumab on December, 2020 (Fig. 2b). Alpha-fetoprotein (AFP) and des-gamma-carboxyprothrombin (DCP) levels temporarily decreased but increased again after two courses of administration. Based on these findings, we diagnosed the patient with progressive disease (PD) and terminated atezolizumab and bevacizumab therapy. Subsequently, cabozantinib treatment (60 mg orally once daily) was initiated on January, 2021. Although liver enzymes were not elevated before the initiation of cabozantinib therapy, grade 3 increases in aspartate aminotransferase and alanine aminotransferase levels (386 IU/L and 105 IU/L, respectively) and anorexia, and grade 1 nausea and vomiting (according to the Common Terminology Criteria for Adverse Events [CTCAE] version 5.0) were observed 1 week after the start of cabozantinib treatment (Table 1). Contrast-enhanced CT showed attenuation of enhancement in the liver lesion and peritoneal dissemination (Fig. 2c), and tumor markers (including AFP and DCP) decreased (Fig. 3, Table 2). However, the decrease in performance status was significant even after treatment interruption, and patients with grade 3 anorexia did not recover. Therefore, we thought that these symptoms resulted from disease progression, and he received only the best supportive care thereafter. His physical condition and activities of daily living had worsened, making it difficult for him to visit the hospital. Finally, the patient and his family opted for home care.

**Table 1:** Laboratory findings after cabozantinib administration

	Day 1	Day 8	Day 15	Day 19	Day 26	Day 140
Alb (g/dL)	3.6	3.4	3.1	3.2	3.2	4.1
T-Bil (mg/dL)	0.8	0.8	0.9	0.9	1.2	0.4
LDH (U/L)	367	1221	862	810	506	183
AST (U/L)	49	386	238	186	82	19
ALT (U/L)	21	65	65	55	34	9
ALP (U/L)	560	647	563	565	467	330
γ-GTP (U/L)	129	142	108	96	73	44
CRP (mg/dL)	2.58	10.94	7.52	4.71	3.14	0.12
WBC (/µL)	5600	6200	5700	6100	3100	6100

*Cr* creatinine, *CRP* carbohydrate reaction protein

**Table 2:** Tumor markers from admission to follow-up after treatment

Months from initial treatment	0	1	2	3	4	5	9
AFP (ng/mL)	20,179.8	24,796	10,075	30,512	44,318.8	17,132.3	9.2
DCP (mAU/mL)	5780	7590	1690	22,100	34,700	5440	22

Along with continued supportive care at home, the symptoms of anorexia and oral intake gradually improved, as did his physical condition. He visited our hospital in June 2021. Contrast-enhanced CT showed attenuation of blood flow in the intrahepatic lesion and peritoneal dissemination nodules, and the tumor size decreased, leading to a diagnosis of CR according to mRECIST (Fig. 2d). The tumors did not progress until April 2022 without any treatment for HCC; however, partial recurrence with increased blood flow on contrast-enhanced CT was observed under the hepatic capsule, leading to a diagnosis of hepatic local recurrence. As there was no obvious recurrence, except for this lesion, carbon-iron radiotherapy (60 GyE/12 fractions) was initiated from June 2022. He has been free from tumor progression and was reported alive until November 2023 (34 months after CR).

## Discussion

We encountered a patient with advanced HCC who showed a CR with cabozantinib following the sixth treatment immediately after treatment with atezolizumab plus bevacizumab. This is the first study reporting a CR with cabozantinib in advanced HCC.

There are two possible reasons for the remarkable effects of cabozantinib observed in this case. One reason might be genetic alterations. Cabozantinib is a tyrosine kinase inhibitor (TKI) that targets receptor kinases involved in immune regulation, tumor growth, and angiogenesis. Its targets include receptor kinases of the VEGFR, MET, and TAM (TYRO3, AXL, and MER) families [6, 7]. Several case reports have reported the remarkable effectiveness of cabozantinib when C-MET gene amplification was observed [8]. Unfortunately, we did not perform a liver biopsy or obtain specimens from recurrent tumors; therefore, genetic testing was not performed in this case. Liver tumor biopsy is not mandatory for diagnosing HCC, and aggressive surgery is often avoided owing to complications such as bleeding, dissemination, and decompensation of the liver function reserve. However, evaluating gene alterations using next-generation sequencing (NGS) can help predict the efficacy of cabozantinib, and tissue sampling and NGS may be encouraged for advanced HCC in the future [8]. The second reason may be that cabozantinib has immunomodulatory activity. HCC is also associated with immune tolerance [9]. This may be linked to the overexpression of cytokine pathways in the tumor microenvironment. Overexpression of cytokine pathways may result in the inhibition of cells associated with the immune response and recruitment of immunosuppressive cells [10]. VEGF overexpression is frequently observed in HCC. Inhibition of T cell function and dendritic cell differentiation, and increased regulatory T cells and myeloid-derived suppressor cells can limit antitumor immune responses [10, 11]. Thus, combining immune checkpoint inhibitors with VEGF-pathway inhibitors and other immunomodulatory pathways may promote an immune-tolerant environment and enhance immune checkpoint inhibitor responses. Moreover, combining cabozantinib and atezolizumab shows efficacy compared to sorafenib monotherapy in the COSMIC-312 trial, and one patient achieved CR [12]. In the CheckMate 9ER trial, the combination of cabozantinib and nivolumab showed better efficacy in renal cell carcinoma than sunitinib alone [13]. These results indicate the efficacy of combination therapy with cabozantinib and an immune checkpoint inhibitor. The efficacy and safety of cabozantinib in patients previously treated with ICI-based therapy have been confirmed in various studies [14]. Although cabozantinib was not concurrently used with atezolizumab in the current case, the effect of atezolizumab may have persisted at the time of cabozantinib initiation since nivolumab binds to T cells over 20 weeks after administration [15].

The possibility of pseudo-progression showing a temporary increase in tumor size owing to immune cell infiltration and a temporary decrease in PS linked to immune-related adverse events should be excluded [16]. In this case, we diagnosed “true” progression against atezolizumab plus bevacizumab since the radiological and laboratory findings indicated disease progression after two courses of treatment.

The present case demonstrated CR according to the mRECIST assessment, and attention should be paid to the fact that the RESIST assessment is a partial response. Although the tumor has shrunken over time, partial recurrence was observed, which required careful follow-up.

Herein, we report a case of advanced HCC that showed a CR with cabozantinib. We hope that more case reports in the future will help elucidate patients that can achieve CR with cabozantinib.
